# Architectural design as a catalyst for healthy aging: An overview of the NCT05031273 study

**DOI:** 10.1192/j.eurpsy.2026.10475

**Published:** 2026-07-31

**Authors:** N. Bouayed Abdelmoula, E. Abdelmoula

**Affiliations:** 1 LR23ES07 Genomics of Signalopathies at the service of Precision Medicine, Medical University of Sfax, Sfax; 2 LR21ES19 UMRAN, National School of Architecture and Urbanism of Tunis; 3 Doctoral school of architectural sciences and engineering (ED-SIA), Carthage University, Carthage, Tunisia

## Abstract

**Introduction:**

The global demographic aging presents urgent challenges for the design of environments that actively support health and well-being later in life. The NCT05031273 clinical trial, conducted by the University of Alberta, is a pioneering initiative that aims to determine if architecture, when intentionally designed to promote movement, nutrition and social connections, can directly influence the lifestyle and health outcomes of older adults.

**Objectives:**

This study aims to present this clinicaltrial as a fundamental model demonstrating how architectural design can function as a form of health intervention for aging populations, and call for the development of similar interdisciplinary studies that unite medical and design expertise using clinicaltrials.gov.

**Methods:**

Using a robust mixed-methods approach, the study combines objective data (accelerometers, GPS, environmental audits, etc.) with in-depth interviews to assess changes in behavior, health and perception after resettlement in housing for the elderly specially designed to promote health.

**Results:**

The study aims to show that the architectural design itself-including walkable layouts, access to communal kitchens and green spaces, natural light and spatial configurations that encourage interaction, plays an essential role in enabling healthier routines. The built typology and characteristics are not simply passive backdrops, but active determinants of behavior, potentially leading to improved levels of physical activity, eating habits, mental wellbeing and social engagement.

**Conclusions:**

This essay marks a turning point in research on aging: recognizing the built environment as a modifiable determinant of health. To translate this knowledge into broader design policies and practices, further trials are needed, based on a strong collaboration between architects, physicians, public health researchers and urban planners. Only through such integration we will be able to develop evidence-based, inclusive and adapted to the realities of aging housing typologies for the elderly.

**Disclosure of Interest:**

None Declared

